# The role of salt abuse on risk for hypercalciuria

**DOI:** 10.1186/1475-2891-10-3

**Published:** 2011-01-06

**Authors:** Patrícia CG Damasio, Carmen RPR Amaro, Natália B Cunha, Ana C Pichutte, José Goldberg, Carlos R Padovani, João L Amaro

**Affiliations:** 1 Graduate Student, Lithotripsy Service, Botucatu School of Medicine, UNESP, Botucatu, Brazil; 2 PhD, Faculty at the Botucatu School of Medicine, UNESP, Lithotripsy Service, Botucatu, Brazil; 3 Undergraduate Student, School of Nutrition, UNESP, Botucatu, Brazil; 4 PhD, Faculty at the Botucatu School of Medicine, UNESP, Department of Urology, Botucatu, Brazil; 5 PhD, Biosciences Institute, Department of Biostatistics, UNESP, Botucatu, Brazil

## Abstract

**Background:**

Elevated sodium excretion in urine resulting from excessive sodium intake can lead to hypercalciuria and contribute to the formation of urinary stones. The aim of this study was to evaluate salt intake in patients with urinary lithiasis and idiopathic hypercalciuria (IH).

**Methods:**

Between August 2007 and June 2008, 105 lithiasic patients were distributed into 2 groups: Group 1 (n = 55): patients with IH (urinary calcium excretion > 250 mg in women and 300 mg in men with normal serum calcium); Group 2 (n = 50): normocalciuric patients (NC). Inclusion criteria were: age over 18 years, normal renal function (creatinine clearance ≥ 60 ml/min), absent proteinuria and negative urinary culture. Pregnant women, patients with intestinal pathologies, chronic diarrhea or using corticoids were excluded. The protocol of metabolic investigation was based on non-consecutive collection of two 24-hour samples for dosages of: calcium, sodium, uric acid, citrate, oxalate, magnesium and urinary volume. Food intake was evaluated by the three-day dietary record quantitative method, and the Body Mass Index (BMI) was calculated and classified according to the World Health Organization (WHO). Sodium intake was evaluated based on 24-hour urinary sodium excretion.

**Results:**

The distribution in both groups as regards mean age (42.11 ± 10.61 vs. 46.14 ± 11.52), weight (77.14 ± 16.03 vs. 75.99 ± 15.80), height (1.64 ± 0.10 vs. 1.64 **± plusorminus **0.08) and BMI (28.78 ± 5.81 vs. 28.07 ± 5.27) was homogeneous. Urinary excretion of calcium (433.33 ± 141.92 vs. 188.93 ± 53.09), sodium (280.08 ± 100.94 vs. 200.44.93 ± 65.81), uric acid (880.63 ± 281.50 vs. 646.74 ± 182.76) and magnesium (88.78 ± 37.53 vs. 64.34 ± 31.84) was significantly higher in the IH group (p < 0.05). There was no statistical difference in calcium intake between the groups, and there was significantly higher salt intake in patients with IH than in NC.

**Conclusions:**

This study showed that salt intake was higher in patients with IH as compared to NC.

## Background

Renal lithiasis is a common disease affecting nearly 20% of the world population, and in approximately 95% of cases, it is associated with a metabolic disorder [1].

Elevated sodium excretion in urine resulting from excessive sodium intake can lead to hypercalciuria and contribute to the formation of urinary stones [2]. Hypercalciuria is the metabolic disorder most frequently found in patients with urinary lithiasis [3,4].

The World Health Organization [5] recommends that the population in general should consume less than 5 grams/day of salt that is 2 g of Na^+^, in order to prevent cardiovascular problems such as arterial hypertension, coronary heart disease and stroke. For lithiasic patients, salt intake should be less than 9 grams/day [6]. However, there are no studies evaluating the isolated role played by sodium restriction in the risk for lithogenesis in patients with hypercalciuria.

Food intake evaluation, and of sodium in particular, will provide information for counseling lithiasic patients, thus enabling individualized treatment in order to prevent stone recurrence in the long term.

The aim of this study was to evaluate salt intake in patients with urinary lithiasis and idiopathic hypercalciuria (IH).

## Methods

From August 2007 to June 2008, 105 lithiasic patients were prospectively studied at the Outpatient Clinic of Renal Lithiasis Metabolism of the Botucatu University Hospital, Unesp. This study was approved by the Bioethics Commission of the School of Medicine - UNESP, Botucatu

Inclusion criteria were: age over 18 years, normal renal function (creatinine clearance ≥ 60 ml/min), absence of proteinuria and negative uroculture at the moment of evaluation. Creatinine clearance was calculated by using the Cockcroft-Gault formula [7]. Pregnant women, patients with intestinal pathologies, chronic diarrhea or using corticoids and diuretics were excluded.

The metabolic investigation protocol consisted of non-consecutive collection of two samples of 24-hour urine for dosages of calcium, sodium, uric acid, citrate, oxalate, magnesium and urinary volume.

The patients were distributed into two groups. Idiopathic hypercalciuria (IH) was considered to be urinary excretion of calcium > 250 mg for women and 300 mg for men with normal serum calcium. Group 1 (n = 55): patients with IH; Group 2 (n = 50): patients with normal urinary excretion of calcium (normocalciuric - NC).

Dietary intake of calcium was calculated by using the three-day dietary record quantitative method and the NutWin software (2002) [8,9]. The measurement of 24-hour urinary sodium excretion is considered to be the gold standard method for data obtained from sodium intakes in population surveys [5]. This method has the advantage of being unaffected by subjective reporting of dietary intakes. Salt intake was estimated by [5]:

$$\text{Salt intake }\left(\text{g}\right)=\frac{{\text{Na}}^{+}\text{ excretion }\left(\text{mEq}/\text{ 24}-\text{hour urine}\right)}{17}$$

(1 g of salt = 17 mEq amount of sodium).

The Body Mass Index (BMI) was calculated and classified according to the World Health Organization [10].

Student's t test was used for independent samples to compare the groups of patients with calcium and non-calcium urinary lithiasis in relation to the quantitative variables studied when the variable presented adherence to the Gaussian distribution; and the Wilcoxon-Mann Whitney non-parametric test was used in cases of non-adherence. Considering the study of the association between pairs of variables, Pearson's linear correlation was used [11]. Relative risk was calculated by multiple logistic-regression analysis [12]. Differences were considered significant for p value < 0.05.

## Results

Among the 105 patients studied, there was homogeneous distribution in both groups regarding mean age, weight, height and BMI. However, it was observed that, on average, according to BMI, patients in both groups were overweight (Table 1).

**Table 1:** Description of the median and standard deviation for age, weight, height and BMI in the different groups studied.

	Group		
		
Studied parameters	IH (n = 55)	NC (n = 50)	P value
**Age **(yrs)	42.11 ± 10.61	46.14 ± 11.52	0.064
**Weight **(kg)	77.14 ± 16.03	75.99 ± 15.80	0.712
**Height **(m)	1.64 ± 0.10	1.64 ± 0.08	0.794
**BMI **(kg/m²)	28.78 ± 5.81	28.07 ± 5.27	0.514
**Males **[n (%)]	22 (40)	22 (44)	0.675

The mean urinary excretion of sodium, uric acid and magnesium was significantly higher in the IH group than in NC (Table 2).

**Table 2:** Description of the median and standard deviation for the biochemical characteristics of 24-hour urine in the different groups studied.

	Group		
		
Urinary Excretion	IH (n = 55)	NC (n = 50)	P value
**Calcium **(mg/24 h)	433.33 ± 141.92	188.93 ± 53.09	< 0.001
**Sodium **(mEq/24 h)	280.08 ± 100.94	200.44 ± 65.81	< 0.001
**Uric acid **(mg/24 h)	880.63 ± 281.50	646.74 ± 182.76	< 0.001
**Magnesium **(mg/24 h)	88.78 ± 37.53	64.34 ± 31.84	< 0.001
**Citrate **(mg/24 h)	563.64 ± 505.45	454.89 ± 361.98	0.211
**Oxalate **(mg/24 h)	34.57 ± 23.41	42.40 ± 28.10	0.122
**Volume **(ml/24 h)	1433.5 ± 474.8	1314.4 ± 392.4	0.166

There was no statistical difference in calcium intake between the groups (Table 3).

**Table 3:** Description of the median and standard deviation for the composition of daily dietary intake in the different groups studied.

	Group		
		
Nutrient	IH (n = 55)	NC (n = 50)	P value
**Calcium **(mg)	520.13 ± 245.62	531.44 ± 299.48	0.832
**Salt **(g)*	16.47 ± 5.93	11.79 ± 3.87	< 0.001

* Based on 24-hour urine

Significantly higher salt intake was observed in patients with IH than in NC (Table 3). However, no association was found between urinary excretion of sodium and calcium in both groups. In NC, salt intake was higher than the amount recommended by WHO [5].

The logistic regression analysis of risk for hypercalciuria in relation to salt intake showed a chance of hypercalciuria that was 3-fold higher in patients whose salt consumption was > 9 g/day.

## Discussion

Urinary lithiasis affects mainly young people in their reproductive period of life, and its control through eating habit changes is important. Epidemiological studies show that the disease is more prevalent in males [13,14]; however, similar studies to ours do not show such remarkable differences regarding gender [15-17]. Our findings are in accordance with the literature and can be explained by the higher female prevalence in the general population.

There was homogeneous distribution of age, weight, height and BMI in both groups; however, overweight was prevalent in both groups. Other authors described [18-22] increased incidence of lithiasis in either obese or overweight patients of both genders. In our study, it was observed that patients with lithiasis were overweight.

Studies have shown that a moderate reduction of salt intake in hypertensive patients may also decrease urinary salt excretion, blood pressure and kidney stone recurrence [23].

Calciuria induced by increased sodium intake can result from the inhibition of re-absorbency of calcium in the proximal tubule [23,24]. Such calciuria increases nearly 40 mg/100 mEq of supplemental sodium in normal adults' diet, and up to 80 mg in patients with hypercalciuria and urinary stones [25].

Significantly higher salt intake was observed in patients with IH, and if the amount (< 5 g salt/day) recommended by WHO [5] is considered, there was increased salt intake in the NC group, which can be determinant in the genesis of cardiovascular diseases. In a randomized trial, Borghi et al. [26] observed that sodium and animal protein restriction cause reduction in urinary calcium excretion. Curhan et al. [27] observed a significant correlation between salt intake and recurrence of kidney stones in women. In both groups in this study, higher salt intake than the recommended by WHO [5] was observed. On the other hand, other authors [20,28-30] observed increased urinary excretion of calcium related to higher sodium intake in normocalciuric patients, which shows that sodium intake control plays an important role in the genesis of lithiasis. In a randomized control trial, Nouvenne et al. [31] observed that, in some patients, salt intake decrease was associated with a slight reduction in calciuria. This fact can be explained by the fact that there are salt-dependent and salt-independent hypercalciuric patients [32].

This study will allow an effective attitude towards the control of salt intake, particularly in patients with IH. For this group of patients, the fact that increased liquid consumption associated with dietary counseling and drug therapy will provide clinical control of the lithiasic disease is also noteworthy.

## Conclusion

This study showed that salt intake was higher in patients with IH as compared to NC.

## List of Abbreviations

IH: Hypercalciuria; NC: Normocalciuric; ml/min: millimeter per minute; BMI: Body Mass Index; VS-Versus; g: grams; mEq: milliequivalents; WHO: World Health Organization; yrs: years; m: meter; kg: kilograms; m^2^: meter squared; mg/24 h: milligrams per 24 hours; n: number; %: percentage; ml/24 h: millimeter per 24 hours.

## Competing interests

The authors declare that they have no competing interests.

## Authors' contributions

PCGD; CRPRA: assisted with study design; data collection, analysis and interpretation. NBC; ACP: assisted with data collection. JG: assisted with draft manuscript revision. CRP: assisted with statistical analysis. JLA: assisted with method design and manuscript writing. All authors: proofed and approved final manuscript.
