# Correction: The role of salt abuse on risk for hypercalciuria

**DOI:** 10.1186/1475-2891-10-63

**Published:** 2011-06-06

**Authors:** Patrícia Capuzzo Garcia Damasio, Carmen Regina Petean Ruiz Amaro, Natália Baraldi Cunha, Ana Carla Pichutte, José Goldberg, Carlos Roberto Padovani, João Luiz Amaro

**Affiliations:** 1 Graduate Student, Lithotripsy Service, Botucatu School of Medicine, UNESP, Botucatu, Brazil; 2 PhD, Faculty at the Botucatu School of Medicine, UNESP, Lithotripsy Service, Botucatu, Brazil; 3 Undergraduate Student, School of Nutrition, UNESP, Botucatu, Brazil; 4 PhD, Faculty at the Botucatu School of Medicine, UNESP, Department of Urology, Botucatu, Brazil; 5 PhD, Biosciences Institute, Department of Biostatistics, UNESP, Botucatu, Brazil

## Correction

Although the focus of our article in Nutrition Journal [1] reports some novel data and has a different focus compared to our publication in the International Brazilian Journal of Urology [2],  we acknowledge that we have duplicated some text and results and that our Nutrition Journal article reports outcome data from the same study population. We have repeated some parts of the methods section from the International Brazilian Journal of Urology as well as the tables showing demographic characteristics and the biochemical characteristics of 24hr urine in the different study groups. Our data on salt intake regarding patients with urinary lithiasis and the related discussion are novel. We apologise for the inappropriate overlap between our two publications and our lack of transparency about the similarities between the two articles. Since publication of this article [1], it has come to our attention that there is an error in the section discussing assumptions about obesity-related costs. Table 2 is correct, indicating that 36% of the population is misidentified when BMI is considered, but there is a typographical error in the text which reported it as 31%.

## References

[B1] Damasio P Amaro CR Cunha NB Pichutte AC Goldberg J Padovani CR Amaro JL The role of salt abuse on risk for hypercalciuria Nutr J 2011 10 3 10.1186/1475-2891-10-3 21211048 PMC3100242

[B2] Damasio P Amaro CR Berto SJ Cunha NB Pichutte AC Padovani CR Amaro JL Urinary Lithiasis and Idiopathic Hypercalciuria: The Importance of Dietary Intake Evaluation Int Braz J Urol 2010 36 5 557 562 10.1590/S1677-55382010000500005 21044372

